# Bilateral rhythmic stimulation as a possible modulator of meningeal lymphatic flow: a regulatory T cell–centered neuroimmune hypothesis of eye movement desensitization and reprocessing

**DOI:** 10.3389/fnint.2026.1758529

**Published:** 2026-02-18

**Authors:** Ioulia Milovanov

**Affiliations:** Independent Researcher, Tel Aviv, Israel

**Keywords:** autism spectrum disorder, autonomic nervous system, bilateral rhythmic stimulation, cervical lymph nodes, EMDR, meningeal lymphatic system, microglia, neuroimmune interactions

## Abstract

Eye Movement Desensitization and Reprocessing (EMDR) is an established therapeutic intervention for post-traumatic stress disorder and related conditions, yet its neurobiological mechanisms remain incompletely understood. While prevailing models emphasize cognitive processes such as working memory taxation and memory reconsolidation, these accounts may not fully explain the durability and generalization of therapeutic effects. Here, we propose a hypothesis in which bilateral rhythmic stimulation associated with EMDR modulates neuroimmune interactions through state-dependent changes in autonomic balance and meningeal lymphatic dynamics. Within this framework, regulatory T cells are conceptualized as contributors to baseline neuroimmune tone, influencing microglial activation states, synaptic stability, and network-level regulation. By integrating findings from autonomic physiology, lymphatic biology, and neuroimmunology, this hypothesis generates testable predictions linking behavioral interventions to sustained neural and behavioral outcomes. The model is intended to guide future experimental investigation rather than assert definitive causal pathways.

## Introduction

We propose that the therapeutic effects of Eye Movement Desensitization and Reprocessing (EMDR) may be partially mediated by bilateral rhythmic stimulation–induced modulation of neuroimmune interactions. Specifically, rhythmic bilateral somatic input associated with EMDR may influence autonomic balance and meningeal lymphatic dynamics, thereby biasing immune surveillance toward regulatory modes of neuroimmune interaction and modulating microglial activity within the central nervous system. In this framework, regulatory T cells are positioned as contributors to baseline neuroimmune tone, supporting synaptic stability, network recalibration, and sustained behavioral change. This hypothesis integrates behavioral neuroscience, autonomic physiology, lymphatic biology, and neuroimmunology into a unified mechanistic model intended to generate testable predictions rather than assert established causal relationships.

Eye Movement Desensitization and Reprocessing is an evidence-based treatment for post-traumatic stress disorder and related conditions, supported by randomized controlled trials and meta-analyses demonstrating significant and durable symptom reduction (Chen et al., 2014; Moreno-Alcázar et al., 2017). The intervention combines recall of distressing memories with bilateral rhythmic stimulation, typically involving guided eye movements, alternating tactile input, or auditory tones (Shapiro, 2017). Dominant mechanistic accounts emphasize cognitive processes such as working memory taxation and memory reconsolidation, which may reduce the vividness and emotional salience of traumatic memories (Schubert and Lee, 2010). While these models account for important aspects of symptom relief, they may not fully explain the persistence and generalization of therapeutic effects, suggesting that additional biological mechanisms may contribute to long-term outcomes.

Beyond cognitive theories, converging psychophysiological evidence indicates that EMDR is associated with early and reproducible shifts in autonomic balance. Experimental and clinical studies report reductions in heart rate and increases in heart rate variability during EMDR sessions, consistent with enhanced parasympathetic tone and reduced sympathetic arousal (Sack et al., 2008). These autonomic changes often emerge rapidly during bilateral stimulation and may precede overt subjective symptom relief, indicating a foundational physiological component of the therapeutic response. Neurophysiological investigations further demonstrate modulation of limbic and associative cortical networks involved in emotional regulation and stress processing (Pagani et al., 2012), supporting the view that EMDR engages distributed brain–body regulatory systems rather than operating exclusively through cognitive mechanisms.

Autonomic state is increasingly recognized as a regulator of immune and lymphatic function. Parasympathetic activity influences lymphatic vessel contractility, permeability, and fluid clearance, thereby shaping immune cell trafficking and tissue homeostasis (Hladky and Barrand, 2014). Accordingly, parasympathetic shifts observed during EMDR may indirectly affect meningeal lymphatic dynamics by altering vascular tone, tissue compliance, and rhythmic physiological oscillations that drive cerebrospinal fluid–lymphatic exchange. Within the present hypothesis, autonomic modulation is conceptualized as an intermediate mechanism linking bilateral rhythmic stimulation to downstream neuroimmune effects.

Functional meningeal lymphatic vessels constitute a critical anatomical and physiological interface between the central nervous system and the peripheral immune system. These vessels facilitate drainage of cerebrospinal fluid, solutes, and immune cells from meningeal compartments toward deep cervical lymph nodes, contributing to immune surveillance and central nervous system homeostasis (Louveau et al., 2015; Aspelund et al., 2015). Meningeal lymphatic function is sensitive to biomechanical and physiological factors including arterial pulsatility, intracranial pressure oscillations, respiration, posture, and head–neck movements (Hladky and Barrand, 2014). Recent evidence underscores that cerebrospinal fluid flow is significantly driven by arterial pulsations, suggesting that rhythmic physiological inputs can directly modulate clearance dynamics (Mestre et al., 2018). Imaging studies in humans further support the relevance of these pathways for cerebrospinal fluid clearance and central nervous system–periphery communication (Ringstad and Eide, 2018).

Deep cervical lymph nodes serve as a major immunological hub for central nervous system–associated antigens and immune cell populations, including regulatory immune subsets capable of participating in immune surveillance (Rustenhoven and Kipnis, 2019). Although direct behavioral modulation of immune cell trafficking has not been demonstrated, well-established mechanical and physiological influences on lymphatic flow support the plausibility that state-dependent changes in lymphatic dynamics may bias immune surveillance patterns.

Regulatory T cells play a central role in maintaining immune homeostasis within the central nervous system, extending beyond suppression of overt inflammation. In addition to limiting excessive immune activation, regulatory immune activity contributes to continuous immune surveillance and fine-tuning of neuroimmune interactions under physiological conditions, including modulation of microglial activation states (De Giacomo and Farez, 2021; Rustenhoven and Kipnis, 2019). Through cytokine-mediated and contact-dependent mechanisms, regulatory immune activity constrains excessive microglial reactivity and promotes phenotypes associated with synaptic stability.

Microglia are key effectors of synaptic refinement and activity-dependent circuit remodeling throughout development and adulthood (Schafer et al., 2012). While tightly regulated microglial pruning is essential for normal brain function, chronic or dysregulated activation can lead to aberrant synaptic elimination, network instability, and behavioral alterations (Zhan et al., 2014). Molecular markers such as transmembrane protein 119 and purinergic receptor P2RY12 have facilitated characterization of microglial states associated with homeostasis versus inflammatory activation (Bennett et al., 2016). Modulation of microglial tone by regulatory immune mechanisms therefore represents a plausible pathway through which neuroimmune balance may influence long-term neural and behavioral outcomes.

Within this framework, bilateral rhythmic stimulation is conceptualized as a patterned somatic input capable of engaging autonomic and biomechanical pathways along the head–neck axis. By shaping autonomic balance and lymphatic dynamics, such stimulation may bias neuroimmune interactions toward regulatory surveillance and microglial homeostasis. Specific applications of bilateral stimulation, including mastoid-targeted approaches, are discussed as hypothesis-consistent extensions rather than foundational elements of the proposed mechanism.

Based on these converging lines of evidence, we propose an integrative mechanistic model linking bilateral rhythmic stimulation to autonomic modulation, meningeal lymphatic dynamics, regulatory immune surveillance, and sustained behavioral and emotional regulation.

## Integrative mechanistic model

**FIGURE 1:**
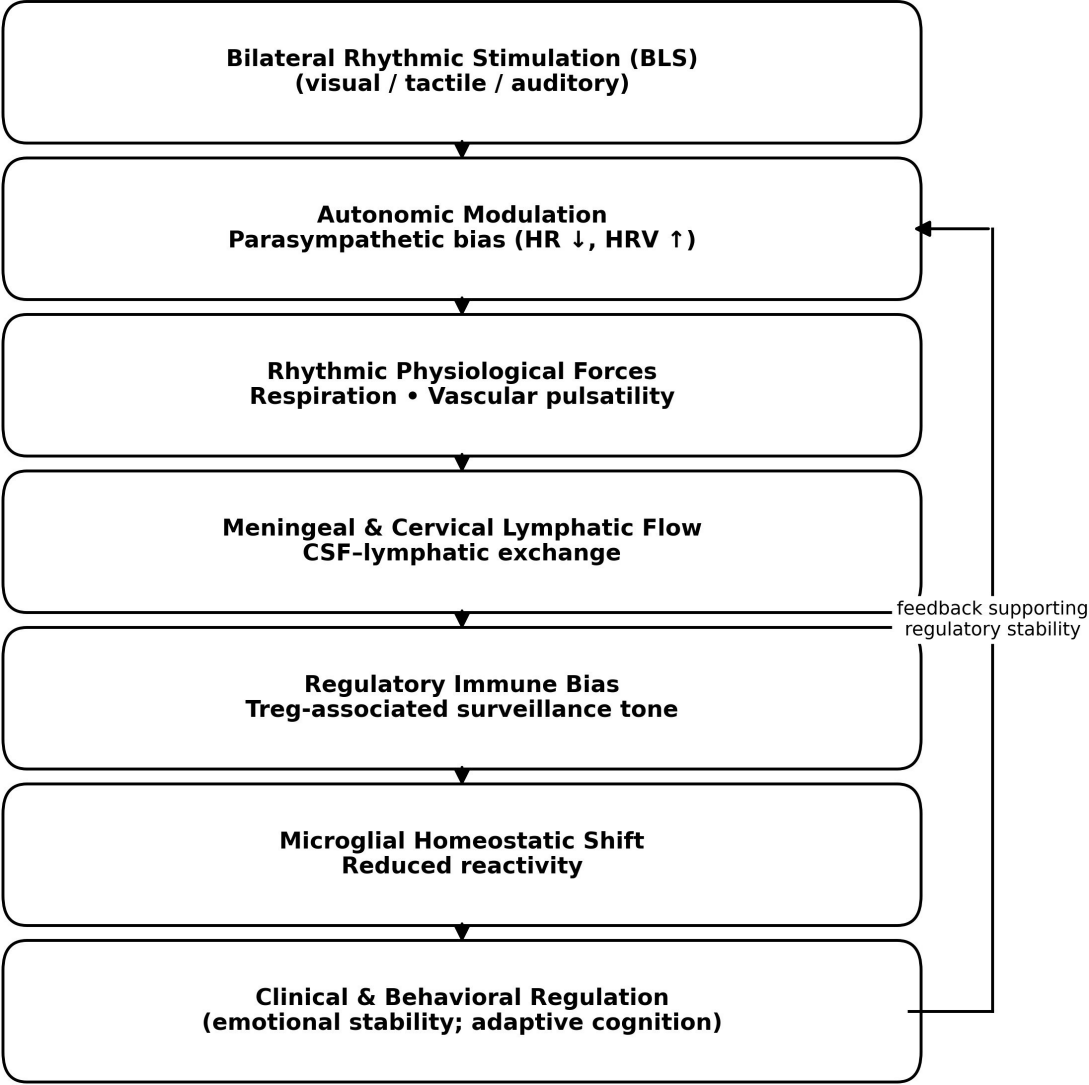
Proposed mechanistic framework linking bilateral rhythmic stimulation to neuroimmune modulation. Bilateral rhythmic stimulation associated with Eye Movement Desensitization and Reprocessing (EMDR) is hypothesized to bias autonomic regulation toward parasympathetic dominance, reflected in reduced sympathetic arousal and increased heart rate variability. These state-dependent autonomic shifts may influence physiological and biomechanical factors that contribute to meningeal and cervical lymphatic dynamics and cerebrospinal fluid–lymphatic exchange. Modulation of lymphatic flow may, in turn, bias immune surveillance toward regulatory modes of interaction, influencing microglial activation states and supporting synaptic and network stability. Behavioral and emotional regulation may further reinforce autonomic balance, forming a feedback loop that supports sustained regulatory stability. This diagram represents a hypothesis-generating conceptual model and does not imply exclusive or obligatory causal pathways.

Based on the converging lines of evidence outlined above, we propose an integrative mechanistic framework linking bilateral rhythmic stimulation to sustained neuroimmune and behavioral effects (Figure 1). This framework is intentionally formulated as a hypothesis-generating model rather than a definitive causal pathway and emphasizes state-dependent modulation of physiological systems over direct immune activation.

Within this model, bilateral rhythmic stimulation associated with EMDR is conceptualized as a patterned somatic input engaging distributed sensory, motor, and autonomic pathways. Empirical studies indicate that such stimulation induces early shifts toward parasympathetic dominance, reflected in reductions in heart rate and increases in heart rate variability, alongside modulation of limbic and associative cortical networks involved in emotional regulation (Sack et al., 2008; Pagani et al., 2012).

The rapid shift toward parasympathetic dominance during bilateral rhythmic stimulation may be further explained through the principle of stochastic resonance. In this context, the rhythmic, low-intensity sensory input associated with Eye Movement Desensitization and Reprocessing may act as a structured signal that enhances the “signal-to-noise ratio” within the autonomic nervous system. By resonating with endogenous physiological rhythms—such as respiratory sinus arrhythmia—this external pacing may facilitate a phase-lock in neural oscillations, effectively “guiding” the system out of a chaotic, high-arousal sympathetic state and into a coherent, homeostatic parasympathetic mode. This autonomic transition, rather than being a mere byproduct, serves as a necessary physiological prerequisite for subsequent shifts in lymphatic and immune dynamics.

These autonomic and neural changes are hypothesized to influence vascular tone, tissue compliance, and rhythmic physiological oscillations that shape cerebrospinal fluid dynamics.

Through combined autonomic and biomechanical pathways, state-dependent physiological changes may modulate meningeal and cervical lymphatic flow. Meningeal lymphatic vessels, which facilitate drainage of cerebrospinal fluid and immune cells toward deep cervical lymph nodes, are sensitive to intracranial pressure oscillations, arterial pulsatility, respiration, and head–neck movements (Hladky and Barrand, 2014; Louveau et al., 2015). Subtle alterations in these parameters may influence lymphatic efficiency and immune surveillance without necessitating overt inflammatory signals.

Within this altered lymphatic milieu, immune surveillance may become biased toward regulatory immune phenotypes rather than effector responses. Regulatory T cells possess migratory and functional characteristics compatible with homeostatic surveillance, including responsiveness to chemokine gradients and the capacity to operate under low-inflammatory conditions (Rustenhoven and Kipnis, 2019; De Giacomo and Farez, 2021). The proposed framework does not imply induction of regulatory T cells *per se*, but rather a shift in relative immune dynamics and local immune tone.

Once present within meningeal or perivascular compartments, regulatory immune activity may influence microglial activation states through cytokine-mediated and contact-dependent mechanisms. Such modulation constrains excessive microglial reactivity and favors phenotypes associated with synaptic stability and network maintenance (Bennett et al., 2016; Schafer et al., 2012). Through these interactions, immune regulation may indirectly shape synaptic refinement and circuit-level organization.

At the systems level, reduced microglial reactivity and improved synaptic stability may contribute to durable changes in emotional regulation, cognitive flexibility, and stress responsiveness. Importantly, these neural and immune effects may, in turn, reinforce autonomic balance by attenuating chronic stress signaling, thereby forming a neuroimmune–autonomic feedback loop. This bidirectional coupling provides a potential explanation for the persistence and generalization of therapeutic effects observed following EMDR and related bilateral stimulation paradigms.

### Clinical and observational correlates

Clinical observations derived from non-controlled therapeutic settings do not establish causality; however, they may reveal recurring patterns consistent with the proposed mechanistic framework and help contextualize its biological plausibility.

### Autism spectrum disorder

Autism spectrum disorder is increasingly conceptualized as involving persistent dysregulation of autonomic and neuroimmune systems, alongside altered neural network development. In therapeutic practice, bilateral rhythmic stimulation has been associated with gradual improvements in regulatory domains, including sensory tolerance, emotional stability, attention, and adaptability to environmental demands. In addition to these regulatory changes, secondary improvements in cognitive and communicative domains—such as language use and social engagement—are frequently observed. Within the proposed framework, such cognitive gains are conceptualized as downstream consequences of improved regulatory and neurophysiological stability rather than as direct targets of the intervention. This interpretation aligns with evidence linking altered immune regulation and microglial activation to atypical synaptic pruning and network instability in autism spectrum disorder (Ashwood et al., 2011; Ahmad et al., 2017; Zhan et al., 2014). Although immune mechanisms cannot be inferred directly from clinical observation, the convergence of behavioral regulation and known neuroimmune features of autism supports the plausibility of the proposed model.

### Stress-related conditions and post-traumatic stress disorder

Chronic stress exposure and post-traumatic stress disorder are associated with sustained autonomic imbalance, reduced parasympathetic tone, and persistent low-grade inflammation (Passos et al., 2015; Michopoulos et al., 2017). In clinical settings utilizing EMDR and related bilateral stimulation paradigms, rapid shifts toward parasympathetic dominance are commonly observed, often preceding subjective reductions in distress (Sack et al., 2008). Over time, these state changes may consolidate into more stable patterns of emotional regulation and stress tolerance. At the neuroimmune level, stress-related disorders have been associated with altered immune regulation and microglial activation, which may contribute to synaptic and network-level changes underlying persistent symptoms (Calcia et al., 2016; Dhabhar, 2014). The proposed framework suggests that bilateral rhythmic stimulation may indirectly influence these processes by engaging autonomic and lymphatic pathways that support regulatory stability, rather than by directly targeting inflammatory mechanisms.

## Experimental and methodological implications

The proposed framework yields experimentally testable predictions across autonomic, lymphatic, immune, and neural domains. The model emphasizes state-dependent modulation rather than direct immune activation, rendering it amenable to non-invasive or minimally invasive experimental approaches. In human studies, central immune dynamics will likely be inferred indirectly through peripheral immune markers and non-invasive neuroimaging-based proxies, reflecting current methodological constraints. In animal models, more direct manipulation of autonomic tone, lymphatic pathways, and immune function may clarify causal relationships among the proposed components. An additional boundary condition concerns the integrity of cervical lymphatic drainage pathways. In individuals with surgically disrupted or compromised cervical lymph nodes, meningeal lymphatic outflow may be altered or rerouted through alternative lymphatic or venous pathways.

Within the proposed framework, such conditions would be expected to attenuate or modify lymphatic contributions to neuroimmune modulation, without precluding autonomic or other regulatory mechanisms. Examination of such populations may therefore help delineate the relative contribution of lymphatic versus non-lymphatic pathways. Similarly, evidence that bilateral rhythmic stimulation–associated immune or microglial changes occur in the absence of concurrent autonomic or lymphatic modulation would prompt reconsideration of the specific mechanistic pathway proposed here, without excluding alternative routes of neuroimmune regulation.

## Conclusion and future directions

This article advances a neuroimmune hypothesis in which bilateral rhythmic stimulation, as employed in EMDR, may exert sustained effects through state-dependent modulation of autonomic balance, meningeal lymphatic dynamics, and regulatory immune surveillance. By integrating emerging insights from meningeal lymphatic biology with established findings in autonomic physiology and neuroimmunology, the framework provides a biologically plausible link between behavioral interventions and durable changes in neural network stability and emotional regulation. The hypothesis is formulated to be empirically testable and open to revision. It predicts coordinated, state-dependent changes across autonomic, lymphatic, immune, and neural domains following bilateral rhythmic stimulation, while explicitly allowing for alternative regulatory pathways. Future research should prioritize multimodal and longitudinal designs integrating autonomic monitoring, non-invasive imaging of lymphatic dynamics, immune profiling, and neural outcome measures. By articulating testable pathways and clear boundary conditions, this framework aims to stimulate interdisciplinary investigation and contribute to a more integrated understanding of how therapeutic interventions can engage endogenous regulatory systems to support long-term neural and behavioral stability.

## Data Availability

The original contributions presented in this study are included in this article/supplementary material, further inquiries can be directed to the corresponding author.
